# FAM83D acts as an oncogene by regulating cell cycle progression via multiple pathways in synovial sarcoma: a potential novel downstream target oncogene of anlotinib

**DOI:** 10.1007/s12672-024-00943-z

**Published:** 2024-03-21

**Authors:** Zi-mei Liu, Ying Yuan, Lei Jin

**Affiliations:** 1 Department of Oncology, Tongren Hospital, Shanghai Jiao Tong University School of Medicine, Shanghai, 200336 China; 2 Department of Rheumatology and Immunology, Tongren Hospital, Shanghai Jiao Tong University School of Medicine, Shanghai, 200336 China; 3 Department of Rheumatology, Immunology & Allergy, Shanghai General Hospital, Shanghai Jiao Tong University School of Medicine, Shanghai, 200080 China

**Keywords:** Anlotinib, Synovial Sarcoma, FAM83D, Proliferation, Apoptosis

## Abstract

**Objective:**

Synovial Sarcoma (SS), a highly malignant mesenchymal neoplasm, typically carries a grim prognosis for patients presenting with high-grade or metastatic disease. Although Anlotinib, a new agent for treating soft tissue sarcomas, holds promise, its underlying mechanism remains incompletely understood. This investigation aims to delineate Anlotinib’s anticancer effectiveness and potential mechanistic underpinnings in patients suffering from advanced, refractory SS.

**Materials and methods:**

Employing microarray assay, we examined the potential downstream targets of Anlotinib in SS therapy. A shRNA-based high-content screening was performed to identify candidate genes with the greatest influence on SW982 cell proliferation. The knockdown efficacy of selected genes within SW982 cells was confirmed using RT-qPCR as well as western blot analysis. To assess the effect of putative downstream elimination of genes with synovial sarcoma cells, cell proliferation, and apoptotic assays were carried out. Gene chip microarray as well as bioinformatics techniques were utilized to scrutinize potential signaling networks associated with the candidate downstream gene.

**Results:**

QPCR verified high expression of FAM83D in SW982 cells, shRNA was designed to silence FAM83D by lentivirus transfection, apoptosis assay, and cell cycle arrest showing that FAM83D downregulation augments apoptosis in SW982 cells and arrests cell cycle progression in the S stage. Inhibition of FAM83D expression upregulated STAT1 while downregulated BIRC5, MCM2, and CDK1 genes in vitro.

**Conclusions:**

This experimental study identified FAM83D as a critical regulator that contributes to the proliferation and progression of SS, suggesting that FAM83D-regulated signaling pathway may serve as a prospective target in SS management.

**Supplementary Information:**

The online version contains supplementary material available at 10.1007/s12672-024-00943-z.

## Introduction

Synovial Sarcomas are a type of malignant tumor of uncertain differentiation, including synovial sarcoma spindle cell, synovial sarcoma biphasic, and synovial sarcoma poorly differentiated. According to the WHO Classification of Bone and Soft Tissue Sarcoma 2020 edition [1], synovial sarcoma is categorized as a type of undifferentiated malignant sarcoma, including subtypes such as fusiform synovial sarcoma, biphasic synovial sarcoma, and poorly differentiated synovial sarcoma. Synovial Sarcoma constitutes a highly aggressive soft tissue malignancy with a poor prognosis, accounting for between five and ten of all soft tissue sarcomas [2]. Existing therapy regimens for SS, encompassing radical surgical intervention and chemoradiotherapy, have yet to demonstrate considerable improvements in prognostic outcomes [3]. Over half of the SS cases are linked with local recurrence or metastatic progression, predominantly afflicting the lungs and bones [4]. In addition, patients frequently become desensitized and unresponsive to chemotherapy and radiotherapy [5]. The 5-year overall survival for advanced SS is about 10% [2]. Therefore, more effective clinical treatments for advanced SS are needed.

Receptor tyrosine kinases (RTKs) have been previously reported to regulate tumor proliferation, growth, angiogenesis, and metastasis, thereby serving important roles in tumor progression [6, 7]. Therefore, targeting RTKs may be an effective strategy for clinical treatment of a variety of solid tumors [8]. Previous clinical evidence demonstrates the efficacy of trabectedin in treating l-sarcomas as well as in non-l-sarcomas, including undifferentiated pleomorphic sarcoma (UPS) [9–11]. Alessandro De Vita et al. [11] have demonstrated that trabectedin exerts cytotoxic effects through the extracellular matrix.

Anlotinib is a novel multi-target RTK inhibitor that has been reported to effectively exert anti-tumor activity among patients presenting with advanced, therapy-resistant solid tumors [12]. Anlotinib targets a range of proteins, such as Vascular Endothelial Growth Factor Receptors 1/2/3 Kit, Platelet-Derived Growth Factor Receptor-α, Discoidin Domain Receptor 1, and Fibroblast Growth Factor Receptors 1/2/3 [13, 14]. As evinced by clinical research, it exhibits a widespread inhibitory influence on neoplastic progression as well as angiogenesis [12, 15] gaining approval for advanced SS therapy by the Chinese Society of Clinical Oncology in 2019 (NCT02449343 as well as NCT01878448) [13, 16]. Moreover, we noted that similar multi-targeted Receptor Tyrosine Kinase Inhibitors (such as Sunitinib, Pazopanib, as well as Sorafenib) with comparable conventional anticancer molecular mechanisms could not match Anlotinib’s anticancer ability inside SS. Consequently, the precise mechanistic action of Anlotinib in advanced SS remains unclear, leading us to hypothesize that Anlotinib could be obstructing SS via alternate downstream pathways. The possible crucial pathways implicated in the biological processing of Anlotinib-treated SS cells remain unexplored.

The purpose of this research was to assess Anlotinib’s anticancer effects on SS in vitro and to identify possible downstream pathways impacted by Anlotinib.

## Materials and methods

### Cellular cultivation

The SS cellular model SW982 and the Human Embryonic Kidney cellular line 293T were obtained from the Chinese Academy of Sciences Cell Bank (Shanghai, China) for this investigational work. These cell lines were grown in Dulbecco’s Modified Eagle’s Media (Hyclone) supplemented with 10% Fetal Bovine Serum (FBS, Gibco), 100 U/ml Penicillin G, plus 100 mg/ml Streptomycin (Sigma, Shanghai, China). Following that, the cells were incubated at 37 °C in a humid atmosphere with 5% CO_2_. One cellular subset underwent therapy with 5 μM Anlotinib for 48 h (CHIATAI TAIQING, China).

### Microarray and bioinformatics analysis

This segment constitutes the experimental portion of the study. The total RNA extraction from the cells was carried out utilizing the Agilent RNA 6000 Nano Kit (Agilent, US) [17], strictly following the manufacturer’s guidelines. The RNA quality was then meticulously evaluated using the Agilent 2100 Bioanalyzer. Microarray execution engaged the GeneChip® PrimeView™ Human Gene Expression Array (Agilent, US). The Significant assessment of Microarrays (SAM) was conducted employing a linear empirical Bayes distribution model to ascertain the level of significant differences in P-value, while the Benjamini–Hochberg methodology corrected these levels. Data visualization included Heatmap, Scatter Plot, as well as Volcano Plot to highlight differences. In addition, hierarchical cluster assessment was implemented during the data examination stage. Lastly, the main method employed was Ingenuity Pathway assessment (IPA), encapsulating classic pathway assessment, disease and functional assessment, regulatory effect evaluation, and interaction network assessment.

### RT-qPCR assay

Upon reaching an 80 percent density within 6-well plates post lentivirus infection over 6 days, SW982 cells were harvested and thrice rinsed with PBS. Subsequent extraction of total RNAs was performed employing TRIzol reagent (Invitrogen, US), following the manufacturer’s instructions. The reverse transcription process was aided by the Prime ScriptTM RT reagent kit (Takara, China) to procure a single cDNA strand. mRNA quantification was then achieved via a Quantitative Real-Time PCR kit utilizing SYBR master mixture (DRR041B, Takara, China). The PCR reaction mixture (20 μl) included: 10 l SYBR PremixEx Taq (2), 0.8 μl Forward primers (10 μM), 0.8 μl Reverse primers (10 μM), 2 μl cDNA, 0.4 μl ROX Reference Dye (50×), as well as 6.0 μl ddH_2_O. The following procedure parameters were established: 95 °C for five seconds, then 60 °C for thirty seconds (for a total of 40 cycles), followed by a holding period at 4 °C. The 2−ΔΔCT approach determined the fold changes in mRNA expression, with experimental outcomes being assessed in relation to GAPDH normalization. The sequences of the primers are shown in Appendix 1.

### Western blotting procedure

Protein quantification from the SW982 cells was accomplished using the BCA kit (Thermo Fisher Scientific, US). Uniform protein amounts underwent a 10 percent SDS-PAGE separation, following which they were transferred onto the PVDF membranes (Amersham Pharmacia Biotech).

Following a 1-h blocking period with 5% non-fat milk, the membranes underwent incubation in a 4 °C environment overnight with primary antibodies, namely Flag (Sigma, #F1804, diluted 1:2000), GAPDH (Santa Cruz, #sc-32233, diluted 1:2000), STAT1 (ABCAM, #AB3987, diluted 1:200), BIRC5 (ABCAM, #AB469, diluted 1:500), MCM2 (ABCAM, #AB108935, diluted 1:1000)., and CDK1 (CST, #9116, at a 1:500 dilution), Membranes were then subjected to three 10-min washes with TBST. The subsequent step involved the utilization of secondary antibodies, namely Goat Anti-Mouse IgG (Santa Cruz, #sc-2005, diluted 1:2000) and Anti-Rabbit IgG (Santa Cruz, #sc-2004, diluted 1:5000), each applied for a span of two hours at room temperature. Following this, an ECL assay kit was employed as per the manufacturer’s recommendation to identify the band signals on the membranes. Each band’s density was then standardized in relation to the expression of GAPDH. (Details on catalog numbers of antibodies used in all western blots are provided in Appendix 2).

### Lentivirus-infected cells

To optimize gene silencing efficiency, this experiment was designed with three RNA interference targets for 20 differential genes. Three plasmids carrying these distinct targets were mixed in equal proportions for lentiviral packaging (Details on the lentiviral vector are provided in Appendix 3). This approach ensured the knockdown efficiency of target genes in subsequent High Content Screening (HCS) experiments following viral infection of cells. Target cells in the logarithmic growth phase were subjected to trypsinization to create a cell suspension; this suspension (approximately 1500–2500 cells) was seeded into 96-well plates and cultured at 37 °C in a 5% CO_2_ incubator until the cell fusion rate reached about 20–30%. Based on the cell’s MOI value, a calculated volume of virus was added. Cell conditions were monitored 12 h later, and the culture medium was subsequently replaced. Observation of the lentivirus-expressed GFP reporter gene occurred 2–3 days post-infection, with fluorescence rates reaching 70–90%, indicating a successful infection. Cells were then cultured until the fusion rate attained 70–90%, at which point they were collected for further experiments.

### High-content screening and cell growth curve analysis

SW982 cells were planted at a density of 2000 cells per well on a plate with 96 wells, after which cells were infected with lentiviruses for about 2–3 days until the green fluorescent protein (GFP) expression rate reached a confluency of 70–90%, then cells were collected for subsequent tests.

Utilization of the Cellomics ArrayScan System facilitated the detection of cellular proliferation. Following this, Cells were seeded at a density of 2000 cells each well in a 96-well plate. Every cohort was given three duplicates. The ceiling Celigo detected read board once a day for the next 5 days commencing on the second day. By adjusting the input parameters, the Cellomics ArrayScan System picked 4 fields of view for each well. The quantity of green fluorescent cells in each scanning plate was accurately determined, followed by the capture of fluorescent images at varied intervals. Data was accumulated, leading to the creation of a cell proliferation curve spanning a continuous 5-day period, which was subsequently utilized for statistical evaluation.

### Construction of FAM83D knockdown lentivirus

According to the sequence of human FAM83D, Short hairpin RNA (shRNA) and control scrambled shRNA (shCtrl) were designed, the sequences are the following:

shRNA: 5′-CCGGGTTCACGTTGATTGATGGCATCTCGAGATGCCATCAATCAACGTGAACTTTTT-3′, shCtrl: 5′CCGGTTCTCCGAACGTGTCACGTTTCAAGAGAACGTGACACGTTCGGAGAATTTTTG-3′. GV115 vectors (Shanghai Genechem, China) carrying shRNA with AgeI and EcoRI restriction enzyme sites were constructed. ShRNAs were used in combination with the helper plasmids pHelper 1.0 and pHelper 2.0 (Shanghai Genechem, China), were introduced to the HEK293T cells utilizing Lipofectamine2000 reagent (Life Technologies, US), adhering to the manufacturer’s guidelines. Following purification, concentration, as well as assurance of quality, the resultant lentiviruses were kept at − 80 °C. SW982 cells were incubated at 6-well plates when cell density at 4 × 105/well, lentivirus-containing media was added into the SW982 cells with a MOI that equals 20. Three days after infection, while the number of GFP-positive cells under the fluorescence microscope reached 70%, qPCR as well as western blot assessments were conducted to validate the efficiency of the effect.

### Determining cell cycle by flow cytometry

The experiment set two groups: the experimental group’s shFAM83D and the control group’s shCtrl. Each group set three duplications, after infection with lentivirus for 6 days. SW982 cells were digested with trypsin when reached a confluency of 80% then collected with a 5 ml centrifugal tube with cell numbers ≥ 106/tube. Cells harvested were subjected to washing with pre-chilled D-Hanks, followed by fixation using 75 percent of pre-chilled ethanol at 4 °C, persisting for a minimum of one hour. Cells then underwent staining with 800 μl of Propidium Iodide (PI) buffer (P4170, Sigma, US) for an hour at 37 °C in a light-obscure environment. Cell cycles were then analyzed using a FACSCalibur flow cytometer (BD, US). The proportion of cells within varying stages (G1, S, as well as G2/M) was discerned via Cell-Quest software (BD, US).

### Determination of cell apoptosis by flow cytometry

The experiment groups were the same as cell cycles, SW982 cells were infected with lentivirus. Upon reaching 80% confluence, cells from each cohort were harvested, rinsed with pre-chilled D-Hanks as well as 1× binding buffer two times, and finally, 10 L of the Annexin V-APC Apoptosis Detection Kit (88-8007, eBioscience, US) was used to stain the cells in a dark setting at room temperature for ten to fifteen minutes before being subjected to apoptosis analysis on flow cytometry.

### Statistical analysis

Each experimental study was conducted thrice, and all data are represented as Means ± SD. Statistical assessment was conducted utilizing SPSS 13.0, employing Student’s t-test, Fisher’s exact assessments, or an ANOVA with a single way (variance analysis) to evaluate data among cohorts, with P < 0.05 denoting statistical significance.

## Results

### Microarray analysis identified FAM83D as a novel downstream target gene of anlotinib

In our initial study [18], we employed microarray profiling (Affymetrix) to scrutinize a plethora of genes in human SW982 cells exposed to anlotinib (5 μM) for the identification of potential anlotinib targets. Relative to the control cohort, 638 genes exhibited significant alterations in expression levels post-anlotinib therapy. Of these genes expressed differentially, 411 were upregulated, while 227 were downregulated (|logFC| > 1, P < 0.01).

**Fig. 1:**
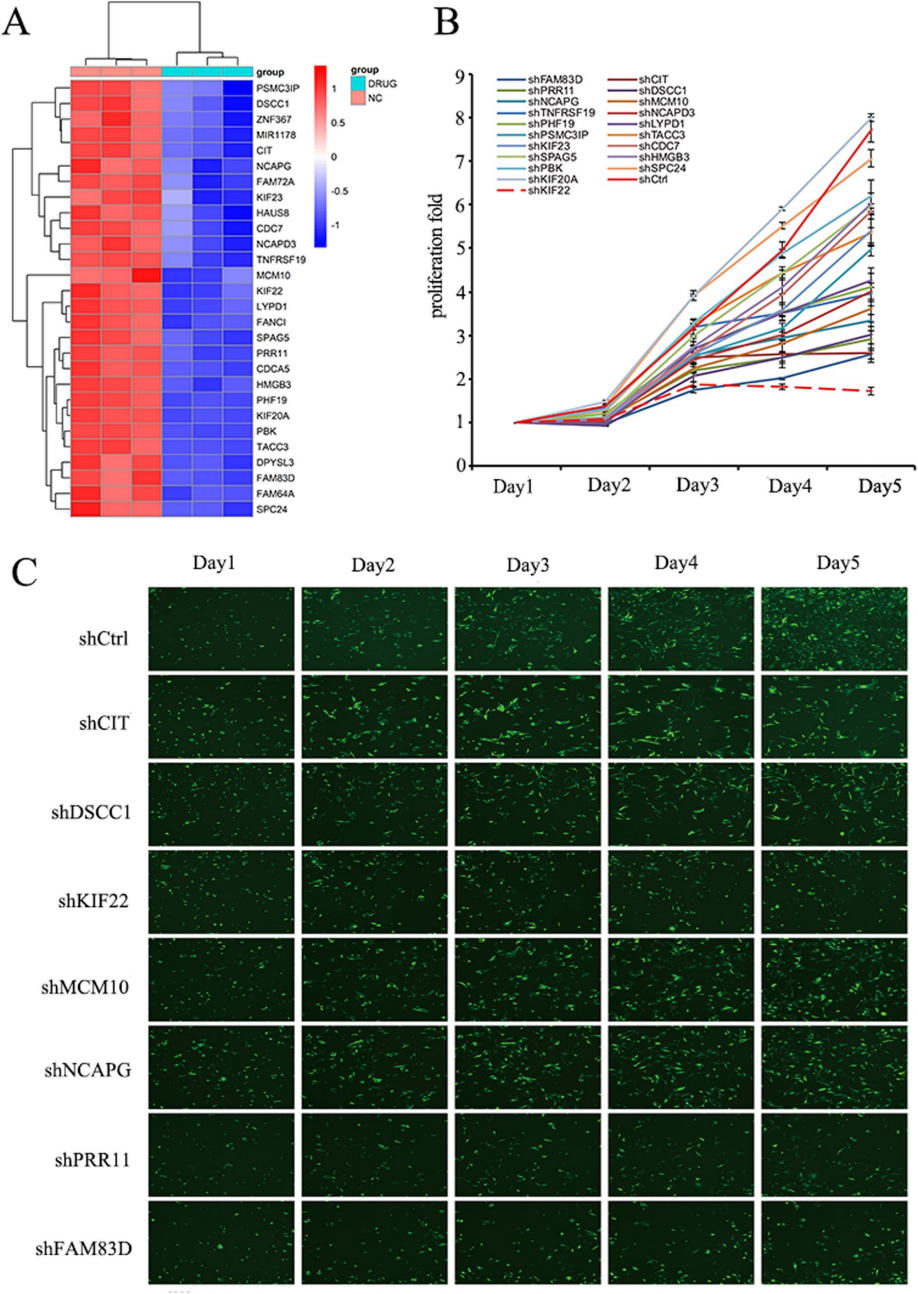
Gene chip microarray and High-content screening identified proliferation-inhibited genes CIT, DSCC1, KIF22, MCM10, NCAPG, PRR11, and FAM83D. **A** Heat map of 28 differential genes after anlotinib was disposed of SW982 cells. **B** Growth curve of 20 genes over 5 consecutive days by high-content screening, Error bars, and standard error. *P < 0.05. **C** Fluorescence images of proliferation-inhibited genes CIT, DSCC1, KIF22, MCM10, NCAPG, PRR11, FAM83D by high-content screening, a fold change of > 2.0 were considered to be significantly inhibited

**Fig. 2:**
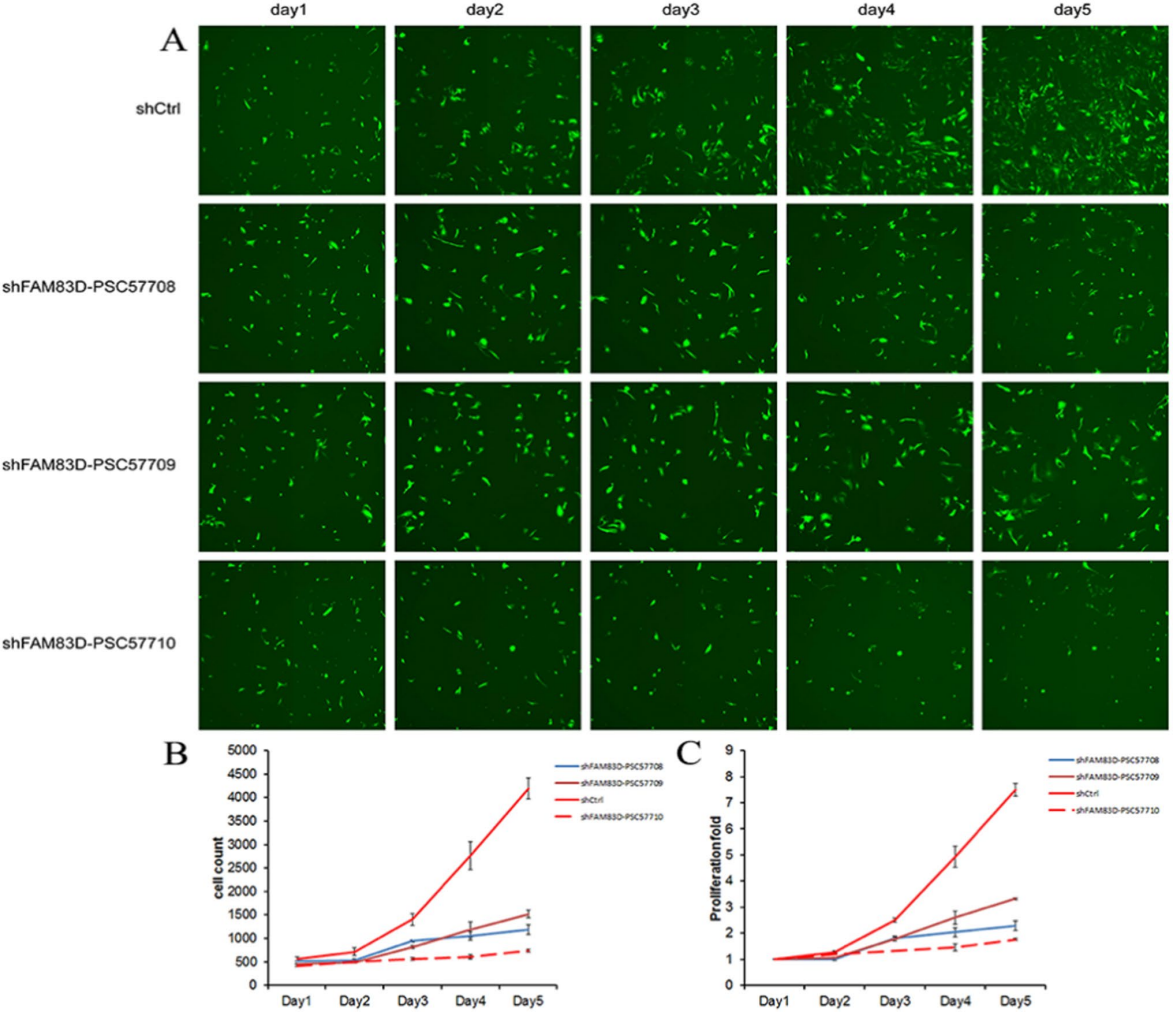
High-content screening identified FAM83D as a vital gene in promoting SS proliferation. **A** Fluorescence images of proliferation-inhibited gene FAM83D by high-content screening Cell count. **B** Cell count, **C** proliferation fold

Among the genes demonstrating downregulation, those genes that changed at least twofold and involved in many signaling pathways were picked as our candidate genes and we focused on 28 candidate genes (Fig. 1A) (Details of 28 candidate genes are provided in Appendix 4 and 5, the false discovery rate (FDR) values for the 28 candidate genes are provided in Appendix 6). QPCR was performed to verify high expression in sw982. Among these genes, the expression abundance of MIR1178 was low in SW982 cells, and the expression abundance of other genes was high and the top 20 genes were selected as the target genes for the experiment (FAM83D, CIT, PRR11, DSCC1, NCAPG, MCM10, TNFRSF19, NCAPD3, PHF19, LYPD1, PSMC3IP, TACC3, KIF23, CDC7, SPAG5, HMGB3, PBK, SPC24, KIF20A, KIF22) (Details on shRNA sequences of selected genes are provided in Appendix 7) (Fig. 1B). To target the 20 aforementioned genes, we engineered 20 lentivirus-mediated siRNAs to silence their expression in SW982 cells. To ascertain the efficiency of gene silencing, 3 targets of RNA interference were devised for each gene, and 3 plasmids that carry varying targets were amalgamated in the same proportions for the packaging of lentivirus, so as to ensure the knockdown efficiency of target genes in subsequent high-content screening assay following virus cell infections. Cells expressing GFP were quantified over five sequential days using the HCS test, following infection with lentivirus-mediated siRNAs including the aforementioned 20 genes or NC-siRNA. SW982 cells expressing GFP were detected over 5 days, and the proliferative activity was evaluated on day 5, considering fold variations in siRNA-treated cohorts compared with the negative control cohort. Genes with a fold change of more than 1.9 and a P-value of less than 0.001 were categorized as differentially expressed. Among the 20 experimental groups to be tested, the experimental group with proliferation multiple change ≥ 2.0 (proliferation inhibition positive cell group), that is, the experimental group with positive proliferation inhibition were: shKIF22, shFAM83D, shCIT, shPRR11, shDSCC1, shNCAPG, shMCM10 (Fig. 1C). Finally, after designing a single RNA interference target for FAM83D and KIF22 to mediate siRNA infection, by conducting high-content screening for 5 uninterrupted days, GFP-expressing cells were enumerated. Our findings indicated a significant curtailment in cellular growth due to FAM83D interference (Fig. 2), signifying the potential role of FAM83D in anlotinib and Synovial sarcoma.

### Silencing of FAM83D expression inhibits SW982 cell growth

**Fig. 3:**
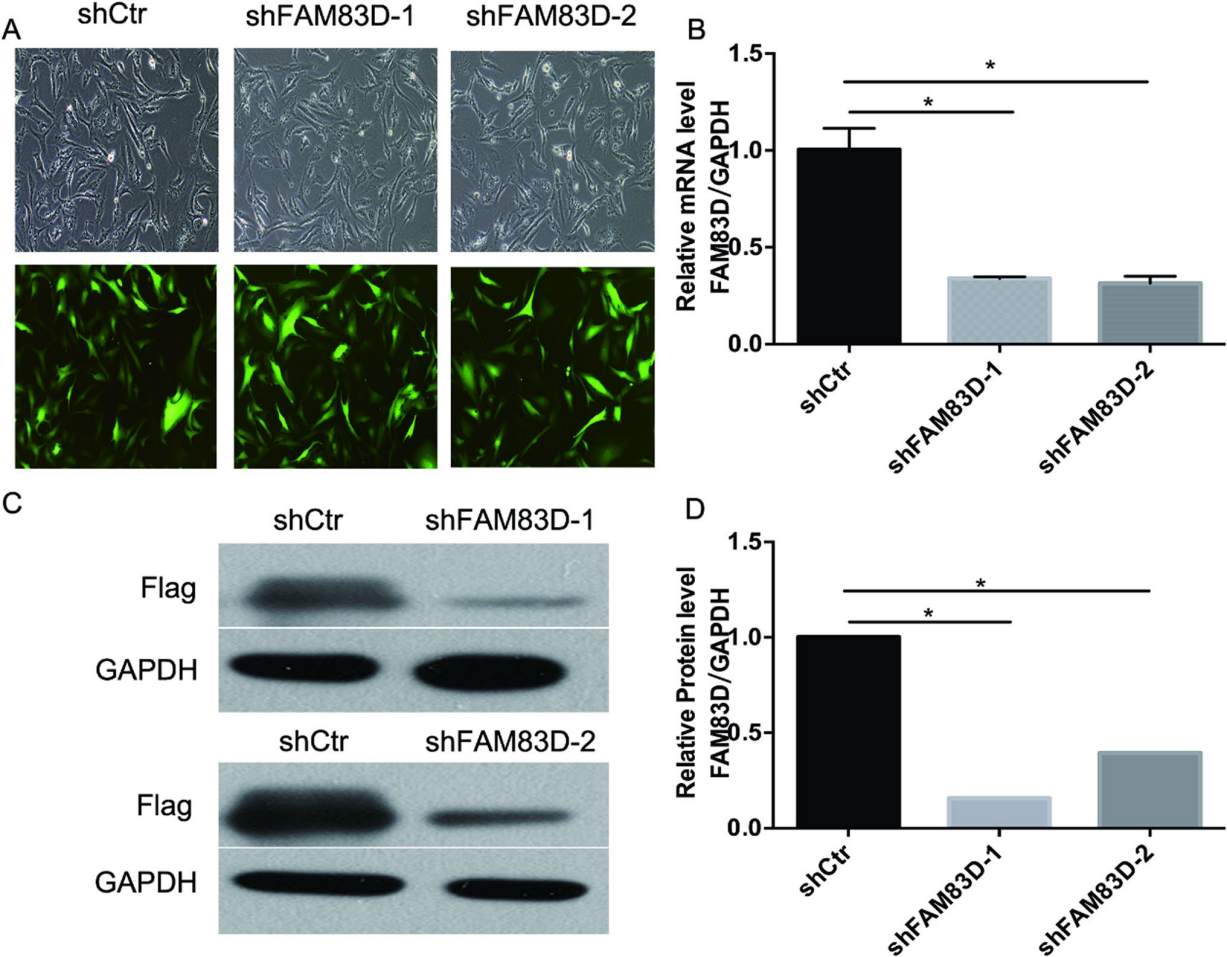
Effects of shRNA mediated FAM83D silence in SW-982 cells. **A** GFP and microscopic image of SW982 cells before and after FAM83D knockdown. **B** QPCR validated FAM83D knockdown efficiency, Error bars, and standard error. *P < 0.05. **C**, **D** Western blot analysis validated the knockdown efficiency of FAM83D in SW-982 cells. *P < 0.05

In the pursuit of discerning the function of FAM83D in Synovial sarcoma, SW982 cell lines were transfected with lentivirus carrying FAM83D shRNA (shFAM83D) as well as control scrambled lentiviral vectors (shCtrl), thereby establishing stable FAM83D silenced cell lines (Fig. 3A). Comparisons of growth conditions involving FAM83D-shRNA or shCtrl-infected SW982 cells as well as non-transfected cells revealed that lentiviral transfection did not influence the growth of cells. Total RNA was extracted and quantified using the real-time polymerase chain reaction method. GINS1 mRNA expression was significantly lower within cells transfected with GINS1 shRNA compared to mock-transfected cells (Fig. 3B). Concurrently, as displayed in Fig. 3C as well as Fig. 3D, FAM83D expression within cells transfected with protein level was diminished in comparison to shCtrl cells.

The impact of FAM83D on the proliferative capability of SW982 cells was evaluated using HCS tests. FAM83D knockdown significantly reduced the growth of SW982 cells, suggesting FAM83D’s role in promoting Synovial sarcoma cell proliferation in vitro.

### The downregulation of FAM83D expression expedited SW982 cell apoptosis as well as prompted cell cycle arrest

**Fig. 4:**
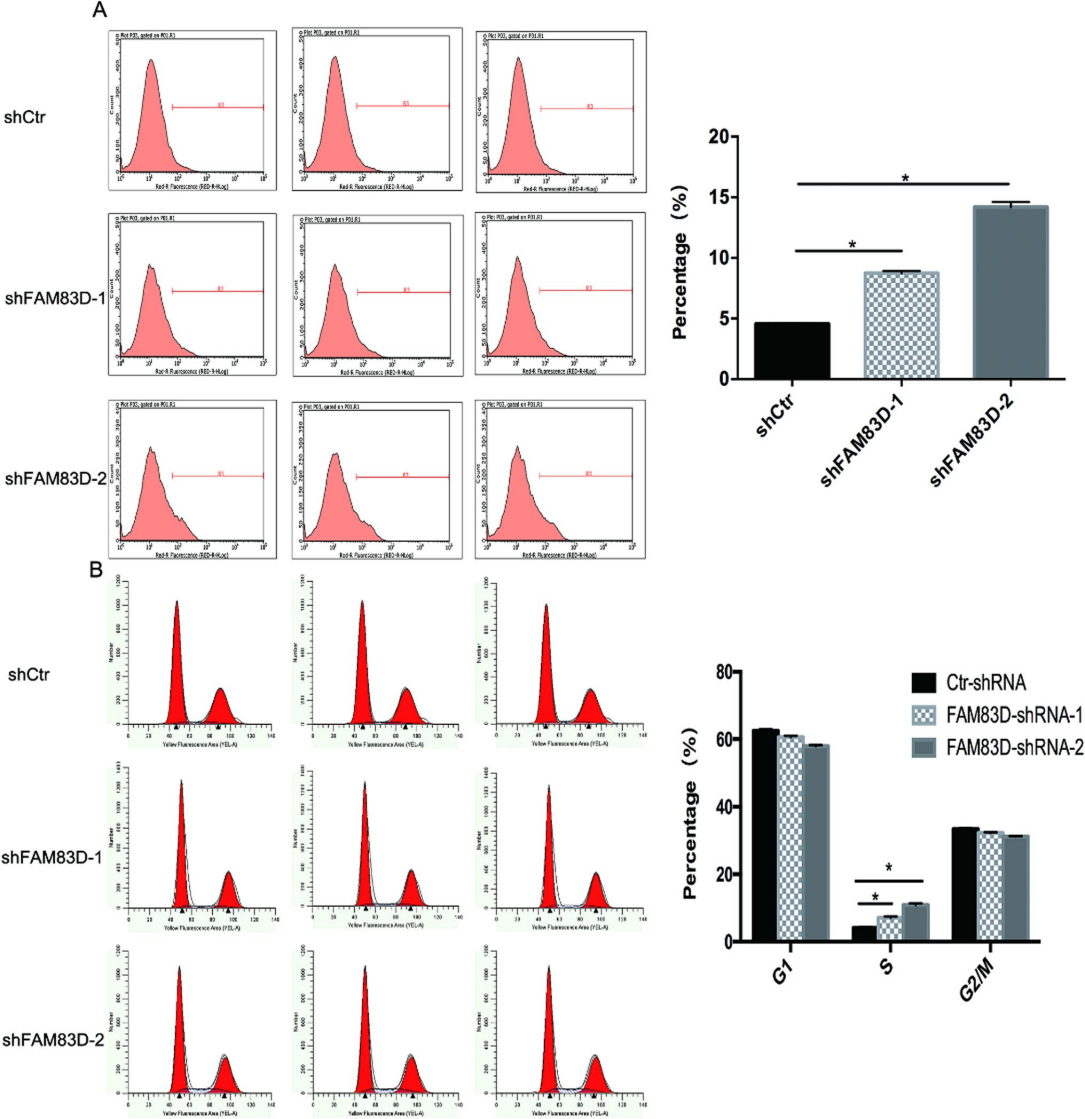
Silencing of FAM83D expression accelerates SW982 cells apoptosis and induces cell cycle arrest. **A** Using FACS to analyze the Apoptosis of two shFAM83D SW982 cell lines and the shCtr cell line, each group designed three biological replicates, Data of graphs are presented as mean ± SD of each independent experiment. Apoptosis significantly increased in FAM83D downregulation cell lines (*P < 0.05). **B** Cell cycle analysis using FACS in mock-transfected and two shFAM83D SW982 cell lines. Graphs presented the percentage of cells in different stages of the cell cycle, and dates were presented as mean ± SD, Error bars, and standard error. *P < 0.05

Cell apoptosis and cell cycle, pivotal in tumor advancement, are notably linked to FAM83D as determined by a flow cytometry test. These apoptotic rates of SW982 cells following FAM83D knockdown were determined using an Annexin V-APC staining. Figure 4A shows that the percentage of apoptotic SW982 cells in shFAM83D is substantially greater in shCtrl (4.23 ± 0.13 versus 14.19 ± 0.42) (*P* equals 0.000). Utilizing the PI single staining assay, SW982 cell cycle dispersion was investigated. The information revealed that the S stage cell population markedly increased (*P* = 0.000) within the shFAM83D cohort, while the G2/M stage significantly dwindled (*P* = 0.000) (Fig. 4B). This suggests that FAM83D could exert a crucial influence on cell apoptosis and S stage cell cycle arrest.

### FAM83D expression suppression inhibits synovial sarcoma through the regulation of STAT1, BIRC5, MCM2, and CDK1

**Fig. 5:**
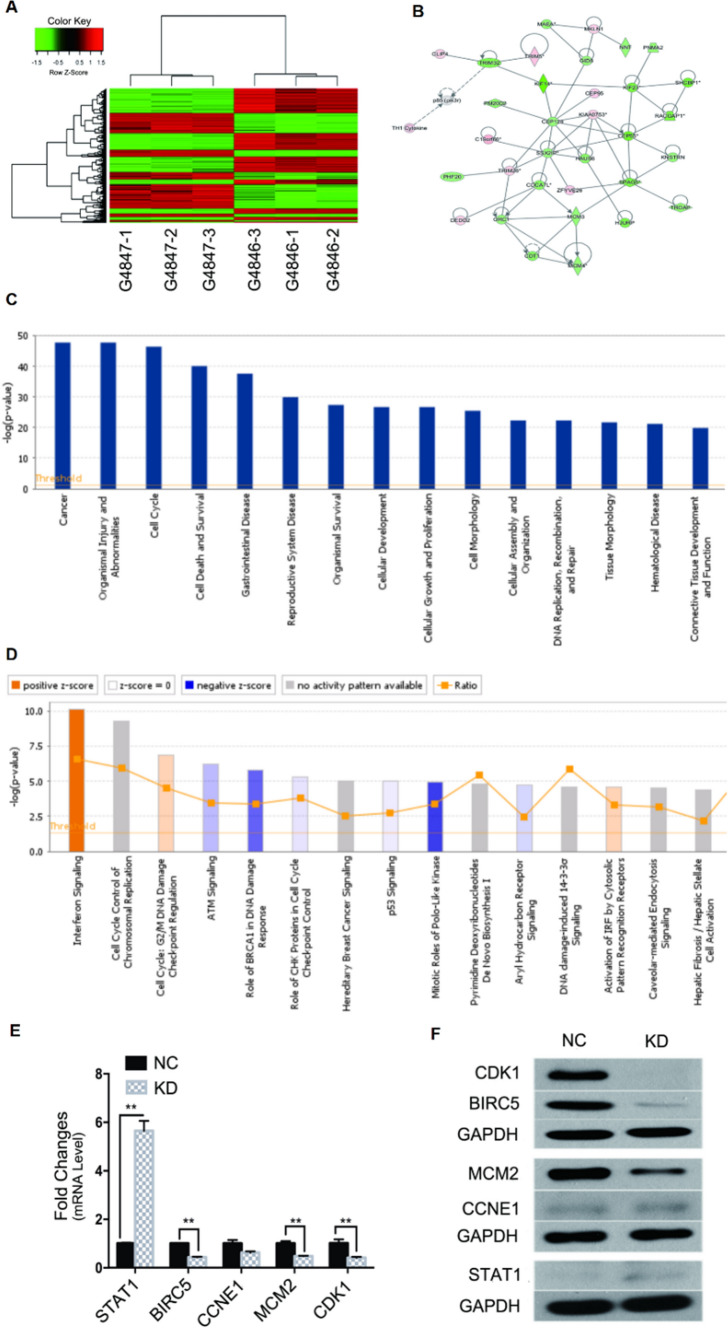
Effects of FAM83D knockdown on the expression of target genes in SW982 cells. **A** Heatmap of microarray gene expression. |Fold Change| > 1.5 and FDR < 0.05 were identified as significant, each row represents a DEG and each column represents a sample. Red indicates high expression, whereas green indicates low expression. *FDR* false discovery rate, *DEG* differentially expressed gene. **B** The network of gene interaction by bioinformatics analyzing. Red indicates upregulation and green indicates downregulation. **C** IPA disease and function analysis. **D** IPA “canonical pathway” analysis. **E** QPCR illustrated the effects of FAM83D silence on mRNA expression. STAT1 was significantly upregulated whereas BIRC5, CCNE1, MCM2, and CDK1 were downregulation, Error bars, and standard error. **P < 0.01, *NC* negative control, *KD* FAM83D knockdown. **F** Western blot presented the effects of FAM83D silence on protein expression. The result coincides with QPCR. *NC* negative control, *KD* FAM83D knockdown

These in vitro results showed that FAM83D is needed for synovial sarcoma growth. The mechanisms underlying FAM83D-mediated synovial sarcoma remain unknown. To comprehensively investigate the downstream pathways, AffymetrixGeneChip and IPA were employed to illustrate the potential biological functionality of FAM83D via FAM83D knockdown within SW982 cells. The heatmap demonstrated that 791 genes were upregulated and 887 were downregulated during FAM83D knockdown cells (Fig. 5A). Selected gene lists from microarray investigations were uploaded into the IPA system, allowing for core biological pathway evaluation and the identification of molecular networks (Fig. 5B). IPA disease/function analysis demonstrated that the diseases “Cancer” and functions “Cell Cycle” and “Cellular Growth and Proliferation” were significantly correlated with downregulated genes (Fig. 5C). The IPA “canonical pathway” module also revealed that multiple critical pathways are involved in cancer development. Within all these pathways, “Interferon signaling”, “DNA Methylation and Transcriptional Repression Signaling” and “Cell Cycle Control of Chromosomal Replication” were activated most significantly, possibly involving anlotinib’s inhibition of Synovial sarcoma proliferation (Fig. 5D).

IPA discovered that various targets that have previously been implicated in the etiology of cancer were differently regulated, such as Signal Transducer as well as Activator of Transcription 1 (STAT1), Baculoviral IAP Repeat-Containing 5 (BIRC5), Minichromosome Maintenance Protein 2(MCM2) and Cyclin-Dependent Kinase 1(CDK1). To substantiate the hypothesized influence of FAM83D on downstream gene regulation, SW982 cells were subjected to GINS1 knockdown. To determine the endogenous expression of the aforementioned four genes, mRNA and protein expression measurements were then performed. As shown in Fig. 5E, F, STAT1 was remarkably upregulated. At the same time, BIRC5, MCM2, and CDK1 were downregulated following FAM83D silencing within SW982 cells. Collectively, these results point to a potential involvement for FAM83D in the development of synovial sarcoma could be mediated via the regulation of STAT1, BIRC5, MCM2, as well as CDK1.

## Discussion

SS is one of soft tissue sarcoma (STS) and the overall survival is not optimistic, especially for patients with metastasis. Today, targeted therapy has been maturely used in epithelium-originated tumors like lung and colorectal cancers. Some multi-targeted drugs like anlotinib and apatinib have been used in STS [19] and received some curative effects, but their mechanisms of it remained unknown.

In this study, through microarray assessment, we discerned significant alterations in FAM83D expression within SW982 cells following therapy with anlotinib. Then we silenced FAM83D using shRNA and studied followed effect on cell growth, apoptosis, and cell cycle. Our result illustrated that the Silencing of FAM83D expression could notably inhibit cell growth, promote apoptosis, and make G1/S phage arrest in SW982 cells. These findings imply that FAM83D, identified as an additional target gene for anlotinib, could offer a potential therapeutic strategy for the treatment of synovial sarcoma.

The gene FAM83D is also referred to as C20orf129, CHICA, and family with sequence similarity 83, member D, is mapped to chromosome 20q. Amplification of such particular areas is commonly observed in diverse kinds of human cancers [20]. FAM83D contains a highly conserved DUF1669 domain in the N terminus, which is vital for the activation of MAPK signaling [21, 22]. Initially, FAM83D was identified as encoding a mitotic spindle-associated protein (MAP) and playing a vital role in mitotic progression. It regulates cell division and chromosome separation equally in the mitotic process [23]. Recently, the role of FAM83D in cancers has been reported in several articles, such as breast malignancy [24], hepatocellular carcinoma [25], gastric cancer [26], in addition to pulmonary adenocarcinoma [27]. According to the research of Yu et al. [28], METTL3 accelerates the tumorigenesis of triple-negative breast cancer by regulating FAM83D m6A modification. Elevated expression of FAM83D is correlated with TP53 mutations and promoter DNA methylation. Network analysis reveals that FAM83D primarily participates in the progesterone-mediated oocyte maturation pathway, cell cycle regulation, and various other signaling pathways [29].

Some researchers also elucidate that FAM83D may demonstrate its bio-function via regulation of mTOR/FBXW7 [30], TP53 [31], and MEK/ERK signaling [22]. However, less definitive evidence of FAM83D has been reported in SS, and the link between FAM83D and SS development is poorly understood.

To address possible downstream cellular mechanisms required for FAM83D, microarray data was analyzed and identified differentially expressed genes affected by FAM83D. After FAM83D downregulation, 791 genes were upregulated and 887 genes were downregulated. IPA and microarray analysis revealed that FAM83D might interact with STAT1, BIRC5, MCM2, and CDK1. These were later confirmed by PCR and Western blot assay, with upregulation of STAT1 and downregulation of BIRC5, MCM2, and CDK1. MCM2 is a gene that is involved in DNA duplication and is needed in the S phase and for cell division and thus contributes to cell apoptosis. Some studies demonstrated that MCM2 can be regarded as similar to ki-67 and thus serves as measuring cancer cell proliferation [32, 33]. BIRC5, a constituent of the Inhibitor of Apoptosis gene lineage, is responsible for encoding proteins that obstruct cellular apoptosis. According to studies, this gene was found to be highly expressed during fetal development as well as in the majority of tumors, while it was shown to be low in adult tissues. As a component of the chromosome passage protein complex, BIRC5 is crucial for chromosome alignment as well as segregation, playing a pivotal role in inhibiting apoptosis and promoting cell proliferation [34]. Some studies found that it interacts with STAT3 and also is an inhibitor of the Caspase apoptosis pathway [35]. The Ser/Thr protein kinase family includes CDK1. A component of the M-stage promoting factor, this protein, has an interaction with cyclins-B and thus is vital in G1/S and G2/M transition [36]. As a Ser/Thr protein kinase, it can phosphorylate MCM2 and BIRC5. To coordinate the many cell cycle phases, CDK1 collaborates with nine different cyclins [37]. STAT1, a STAT family member, responds to cytokines and growth factors, translocating to the cell nucleus to function as a transcription activator [38]. It is involved in the Interferon Signaling pathway and can be activated by EGF, IL6, and PDGF [39]. As our best known, PDGF is a high expression in SS and maybe coexpression with ALK/MET. Studies revealed that PDGFR can through activated STAT1 and other distinct pathways promote mammary cancer metastasis [40].

This study, however, is not without its limitations. In our clinical practice, anlotinib has shown some anticancer efficacy against synovial sarcoma, prompting further investigation into its mechanisms of action and target specificity. Through gene chip analysis and high-throughput screening, potential targets were identified and validated. However, due to constraints in time and funding, the research was limited by a lack of sufficient clinical samples. Guided by the principle of serving clinical needs, future experiments will seek to validate our findings with clinical samples.

## Conclusion

FAM83D could interact with its downstream targets via the regulation of STAT1, BIRC5, MCM2, as well as CDK1. Thus, this research provides partial clarification as to why anlotinib therapy as well as FAM83D silencing can inhibit cell growth as well as induce cell death within SW982 cells, in alignment with clinical research outcomes. Investigations into the molecular mechanisms underpinning FAM83D’s regulation of STAT1, BIRC5, MCM2, as well as CDK1 in synovial sarcoma, are currently underway in our laboratory.

### Supplementary Information

Below is the link to the electronic supplementary material.

Supplementary file 1 (DOCX 30 KB)

Supplementary file 2 (DOCX 796 KB)

## Data Availability

Due to space constraints, the original data cannot be fully reflected in the text. Due to format limitations (the original data is in Excel format), it cannot be uploaded to the submission system as a supplementary file. If necessary, please contact the corresponding author to provide.
