# Supplementary material for: FAM83D acts as an oncogene by regulating cell cycle progression via multiple pathways in synovial sarcoma: a potential novel downstream target oncogene of anlotinib
Source: Discov Oncol. 2024 Mar 21;15:82. doi: 10.1007/s12672-024-00943-z (PMC10957831; doi:10.1007/s12672-024-00943-z)
Supplement: Supplementary file 1 — (DOCX 30 KB) [file 12672_2024_943_MOESM1_ESM.docx]

**Appendix 1. Primers used in the experiment.**

| **Gene** | **Upstream primer (5’-3’)** | **Downstream primer (5’-3’)** |
| --- | --- | --- |
| GAPDH | TGACTTCAACAGCGACACCCA | CACCCTGTTGCTGTAGCCAAA |
| FAM83D | GGAAGTGAGACAGGAGTTTGG | TGACAGTTCGGACTATCACAGG |
| KIF20A | AGGTTCTTGCGTACCACAGAC | TGCTGTCCGATGACGATGTC |
| CIT | CAGGCAAGATTGAGAACG | GCACGATTGAGACAGGGA |
| CDC7 | GTTTCACTGCAGGGCTCTCAT | GGAAAAGACGGAAAGGAGGG |
| DSCC1 | CGCATTCTGAAGTAGCATTCGT | TCAGGAACTCGGACCATTGGA |
| NCAPG | AACTGTCTTATCATCCATCGTGC | GAGGCTGCTGTCGATTAAGGA |
| LYPD1 | CCCGTTACAAAGAGGGGTGTT | TTTTGCGGATTGTTCTTGCTT |
| MCM10 | TTTACAGGTTCCCAGGTCAAG | GAAGAAGGTTACGCCACAGAG |
| PSMC3IP | GCATCTCAGCCTCCATGTAGCG | TGCGGATCAGGACCAGTTTGAC |
| TACC3 | CTGAGAACCAAATGGTGTCTCC | CTGTCCTGCAAGGGTCTTCT |
| PBK | GCAGAAGGACGATCTTTAGGGTC | TACTATGCAGCGTTGGGAACT |
| ZNF367 | TGCATGGGTGAATCTGCTCAG | TGTGTGACTATCCAGACTGTGG |
| SPAG5 | GCTTTCCTTGGAGCAATGTAGTT | TTGAGGCCCGTTTAGATACCA |
| PHF19 | ACTCGGGACTCCTATGGTGC | CCTCCGTCAGTTTGGACATCA |
| PRR11 | GAGATGGTCTTCAGTGCTTCCT | CGTATCTGCCACCGAGAACTT |
| CDCA5 | GGGAGATTCCAGGGAGAGTCAT | AGAAAGTCAGGCGTTCCTACAG |
| FAM64A | ACGGAGGTCCCCATGTTCT | CTCGGCCTTAGTGGTGTCG |
| DPYSL3 | AGCCTCGGCATAGATGGAACC | TTCTGGGCAGTGCTGAAGGTG |
| FAM72A | GCTGGCTGATACTGAAATAGACC | CGTTGTTGCAGGAAAGAAGAC |
| NCAPD3 | TCTAACCGTTCCGAACCCTCA | GGTCTTCTCATCCCTGATCCTC |
| TNFRSF19 | CAGAATGCACCCCACAACCAAG | CTCCACGAATATGCCCACAGAG |
| FANCI | GAGTAAGAGCCTGAACTATACGG | AAAGATGAGGTTAGGGATTGG |
| HAUS8 | CAGGCACTGTCTTGATTGAAATA | TGGGAGAACTTGATGTTGGTG |
| KIF23 | GTCCAAACAACGAGGAAGCAG | GAGGGCTACAGACTCAACCGAA |
| HMGB3 | CAGGAAGAATCCAGACGGT | AATGGCAAAGGCAGATAAAG |
| SPC24 | TCAATGCGAAGGAGCAGGTG | AGGGGATTGTGACTGTCGTGTC |
| MIR1178 | GAGGAAGGGAAGGGTCCAGG | TCTAGGGAAGAACAGTGAGCAA |
| KIF22 | GGACCAAGCAATTCTTTCTGA | CTCCCTCGTGTACCTTATCGG |

**Appendix 2. Details on** **catalog numbers of antibodies used in all western blots.**

| **Antibody** | **Source species** | **Corporation** | **Item number** | **Dilution ratio** | **Molecular weights** |
| --- | --- | --- | --- | --- | --- |
| STAT1 | Mouse | ABCAM | AB3987 | Proper ratio | 91(87)kDa |
| BIRC5 | Rabbit | ABCAM | AB496 | 1ug/ml | 18(16)kDa |
| CCNE1 | Mouse | CST | #4129 | 1/1000 | 48kDa |
| MCM2 | Rabbit | ABCAM | AB108935 | 1/2000 | 125(102)kDa |
| CDK1 | Mouse | CST | #9116 | 1/1000 | 34kDa |
| GAPDH | Mouse | SANTA  CRUZ | Sc-32233 | 1:2000 | 36kDa |

**Appendix 3. Details on the lentiviral vector.**

| **Virus serial number** | **Virus name** |
| --- | --- |
| LVpGCSIL-004PSC57710-1 | LV-FAM83D-RNAi (57710-1) |
| Psc3741 | Negative control virus CON053 |

**Appendix 4.** **Expression abundance of 28 genes** **in SW982**

| **Gene** | **GAPDH** | **Target Gene** | **∆Ct** | **2-∆Ct Target Gene** | **2-∆Ct Internal reference** | **expression** |
| --- | --- | --- | --- | --- | --- | --- |
| CIT | 13.1 | 24.48 | 11.38 | 0.000375214 | 0.000113896 | 3.294364069 |
|  | 13.09 | 24.5 | 11.41 | 0.000367492 | 0.000114688 | 3.20427951 |
|  | 13.04 | 24.41 | 11.37 | 0.000377823 | 0.000118732 | 3.182145935 |
| PBK | 13.1 | 20.58 | 7.48 | 0.005601388 | 0.000113896 | 49.1800058 |
|  | 13.09 | 20.66 | 7.57 | 0.005262631 | 0.000114688 | 45.88656794 |
|  | 13.04 | 20.63 | 7.59 | 0.005190179 | 0.000118732 | 43.71328822 |
| NCAPG2 | 13.1 | 23.7 | 10.6 | 0.000644291 | 0.000113896 | 5.656854249 |
|  | 13.09 | 23.61 | 10.52 | 0.000681027 | 0.000114688 | 5.938094283 |
|  | 13.04 | 23.59 | 10.55 | 0.000667012 | 0.000118732 | 5.617779503 |
| ZNF367 | 13.1 | 24.19 | 11.09 | 0.000458751 | 0.000113896 | 4.0278222 |
|  | 13.09 | 23.75 | 10.66 | 0.000618045 | 0.000114688 | 5.388934307 |
|  | 13.04 | 24.29 | 11.25 | 0.000410594 | 0.000118732 | 3.458148925 |
| SPAG5 | 13.1 | 24.82 | 11.72 | 0.000296434 | 0.000113896 | 2.602683711 |
|  | 13.09 | 24.97 | 11.88 | 0.000265316 | 0.000114688 | 2.313376368 |
|  | 13.04 | 24.84 | 11.8 | 0.000280444 | 0.000118732 | 2.361985323 |
| KIF20A | 13.1 | 22.9 | 9.8 | 0.001121776 | 0.000113896 | 9.849155307 |
|  | 13.09 | 22.69 | 9.6 | 0.001288582 | 0.000114688 | 11.23555901 |
|  | 13.04 | 22.65 | 9.61 | 0.001279681 | 0.000118732 | 10.77786861 |
| PHF19 | 13.1 | 20.81 | 7.71 | 0.004775939 | 0.000113896 | 41.93258892 |
|  | 13.09 | 21.1 | 8.01 | 0.003879268 | 0.000114688 | 33.8245773 |
|  | 13.04 | 20.93 | 7.89 | 0.004215735 | 0.000118732 | 35.50622311 |
| PRR11 | 13.1 | 22.18 | 9.08 | 0.001847769 | 0.000113896 | 16.22335168 |
|  | 13.09 | 22.24 | 9.15 | 0.001760255 | 0.000114688 | 15.34822591 |
|  | 13.04 | 22.24 | 9.2 | 0.001700294 | 0.000118732 | 14.32040113 |
| DSCC1 | 13.1 | 23.21 | 10.11 | 0.000904871 | 0.000113896 | 7.944739963 |
|  | 13.09 | 23.08 | 9.99 | 0.000983355 | 0.000114688 | 8.5741877 |
|  | 13.04 | 22.96 | 9.92 | 0.001032244 | 0.000118732 | 8.6938789 |
| CDCA5 | 13.1 | 23.59 | 10.49 | 0.000695337 | 0.000113896 | 6.105036836 |
|  | 13.09 | 23.51 | 10.42 | 0.000729907 | 0.000114688 | 6.36429187 |
|  | 13.04 | 23.1 | 10.06 | 0.000936781 | 0.000118732 | 7.889861636 |
| FAM64A | 13.1 | 22.14 | 9.04 | 0.001899717 | 0.000113896 | 16.67945217 |
|  | 13.09 | 22.04 | 8.95 | 0.002022002 | 0.000114688 | 17.63048185 |
|  | 13.04 | 22.04 | 9 | 0.001953125 | 0.000118732 | 16.44982123 |
| DPYSL3 | 13.1 | 24.04 | 10.94 | 0.000509016 | 0.000113896 | 4.469148552 |
|  | 13.09 | 24.33 | 11.24 | 0.00041345 | 0.000114688 | 3.60500185 |
|  | 13.04 | 24.05 | 11.01 | 0.000484908 | 0.000118732 | 4.084048503 |
| MCM10 | 13.1 | 22.27 | 9.17 | 0.001736021 | 0.000113896 | 15.24220797 |
|  | 13.09 | 22.24 | 9.15 | 0.001760255 | 0.000114688 | 15.34822591 |
|  | 13.04 | 22.22 | 9.18 | 0.001724029 | 0.000118732 | 14.52030649 |
| LYPD1 | 13.1 | 22.59 | 9.49 | 0.001390674 | 0.000113896 | 12.21007367 |
|  | 13.09 | 22.49 | 9.4 | 0.001480192 | 0.000114688 | 12.90626815 |
|  | 13.04 | 22.28 | 9.24 | 0.001653799 | 0.000118732 | 13.92880901 |
| TACC3 | 13.1 | 22.99 | 9.89 | 0.001053934 | 0.000113896 | 9.253505471 |
|  | 13.09 | 22.8 | 9.71 | 0.001193985 | 0.000114688 | 10.41073484 |
|  | 13.04 | 22.82 | 9.78 | 0.001137435 | 0.000118732 | 9.579829637 |
| FAM72A | 13.1 | 21.76 | 8.66 | 0.002472181 | 0.000113896 | 21.70566924 |
|  | 13.09 | 21.69 | 8.6 | 0.002577164 | 0.000114688 | 22.47111801 |
|  | 13.04 | 21.5 | 8.46 | 0.00283979 | 0.000118732 | 23.91758798 |
| NCAPD3 | 13.1 | 24.53 | 11.43 | 0.000362433 | 0.000113896 | 3.182145935 |
|  | 13.09 | 24.35 | 11.26 | 0.000407758 | 0.000114688 | 3.555370725 |
|  | 13.04 | 24.33 | 11.29 | 0.000399366 | 0.000118732 | 3.363585661 |
| PSMC3IP | 13.1 | 21.88 | 8.78 | 0.00227487 | 0.000113896 | 19.97328878 |
|  | 13.09 | 21.76 | 8.67 | 0.002455104 | 0.000114688 | 21.40684088 |
|  | 13.04 | 22 | 8.96 | 0.002008035 | 0.000118732 | 16.91228865 |
| TNFRSF19 | 13.1 | 24.62 | 11.52 | 0.000340514 | 0.000113896 | 2.989698497 |
|  | 13.09 | 24.33 | 11.24 | 0.00041345 | 0.000114688 | 3.60500185 |
|  | 13.04 | 24.49 | 11.45 | 0.000357443 | 0.000118732 | 3.010493495 |
| CDC7 | 13.1 | 24.47 | 11.37 | 0.000377823 | 0.000113896 | 3.317278183 |
|  | 13.09 | 24.14 | 11.05 | 0.000471649 | 0.000114688 | 4.112455307 |
|  | 13.04 | 24.27 | 11.23 | 0.000416326 | 0.000118732 | 3.506422885 |
| FANCI | 13.1 | 20.82 | 7.72 | 0.004742949 | 0.000113896 | 41.64293937 |
|  | 13.09 | 20.92 | 7.83 | 0.00439476 | 0.000114688 | 38.31931855 |
|  | 13.04 | 20.72 | 7.68 | 0.004876291 | 0.000118732 | 41.06962872 |
| FAM83D | 13.1 | 20.04 | 6.94 | 0.008144264 | 0.000113896 | 71.50637684 |
|  | 13.09 | 20.05 | 6.96 | 0.008032139 | 0.000114688 | 70.03479688 |
|  | 13.04 | 19.83 | 6.79 | 0.009036626 | 0.000118732 | 76.10925536 |
| HAUS8 | 13.1 | 21.33 | 8.23 | 0.003330605 | 0.000113896 | 29.24260641 |
|  | 13.09 | 21.09 | 8 | 0.00390625 | 0.000114688 | 34.05984584 |
|  | 13.04 | 21.14 | 8.1 | 0.00364466 | 0.000118732 | 30.69645182 |
| KIF23 | 13.1 | 21.32 | 8.22 | 0.003353771 | 0.000113896 | 29.44600482 |
|  | 13.09 | 21.08 | 7.99 | 0.00393342 | 0.000114688 | 34.2967508 |
|  | 13.04 | 21.52 | 8.48 | 0.002800694 | 0.000118732 | 23.58830748 |
| HMGB3 | 13.1 | 21.09 | 7.99 | 0.00393342 | 0.000113896 | 34.53530357 |
|  | 13.09 | 21.08 | 7.99 | 0.00393342 | 0.000114688 | 34.2967508 |
|  | 13.04 | 21.07 | 8.03 | 0.003825861 | 0.000118732 | 32.2225776 |
| SPC24 | 13.1 | 23.8 | 10.7 | 0.000601145 | 0.000113896 | 5.278031643 |
|  | 13.09 | 23.76 | 10.67 | 0.000613776 | 0.000114688 | 5.351710219 |
|  | 13.04 | 23.72 | 10.68 | 0.000609536 | 0.000118732 | 5.13370359 |
| MIR1178 | 13.1 | 29.18 | 16.08 | 1.44357E-05 | 0.000113896 | 0.126744935 |
|  | 13.09 | 28.75 | 15.66 | 1.93139E-05 | 0.000114688 | 0.168404197 |
|  | 13.04 | 28.93 | 15.89 | 1.64677E-05 | 0.000118732 | 0.138696184 |
| KIF22 | 13.1 | 19.89 | 6.79 | 0.009036626 | 0.000113896 | 79.3412928 |
|  | 13.09 | 19.67 | 6.58 | 0.010452559 | 0.000114688 | 91.13921252 |
|  | 13.04 | 20.06 | 7.02 | 0.007704943 | 0.000118732 | 64.89340671 |

**Appendix 5. Target information for 28 genes**

| **Clone sequencing number** | **Gene ID** | **Species** | **Genes** | **GenBank_ID** | **Target sequence** |
| --- | --- | --- | --- | --- | --- |
| psc14188-1 | 8317 | Human | CDC7 | NM_003503 | ccTAATCTGTTTGGTAAGTAT |
| psc14190-1 | 8317 | Human | CDC7 | NM_003503 | gcCACAGCACAGTTACAAGTA |
| psc14192-1 | 8317 | Human | CDC7 | NM_003503 | ccAGGACAATACTCAGGGAAT |
| psc13974-1 | 11113 | Human | CIT | NM_007174 | cgGAAGTATTCCGACACCATA |
| PSC2915-1 | 11113 | Human | CIT | NM_007174 | GCGTCCTCATACCAGGATAAA |
| PSC2916-1 | 11113 | Human | CIT | NM_007174 | GCCAATAAACTTGCAGCAAAT |
| PSC29887-1 | 79075 | Human | DSCC1 | NM_024094 | GTAGATGATTTACCTGAGGAT |
| PSC29888-1 | 79075 | Human | DSCC1 | NM_024094 | GCCTGTAAGATTGGAGGTTAT |
| PSC29889-1 | 79075 | Human | DSCC1 | NM_024094 | GAAGACGTAGACCCAAGTTAA |
| PSC57708-1 | 81610 | Human | FAM83D | NM_030919 | GATCTGAAAGTTCATCCTGAA |
| PSC57709-1 | 81610 | Human | FAM83D | NM_030919 | CCTGACTTTGTCACCTTTGTT |
| PSC57710-1 | 81610 | Human | FAM83D | NM_030919 | GTTCACGTTGATTGATGGCAT |
| PSC29474-1 | 3149 | Human | HMGB3 | NM_005342 | GCAGATAAAGTGCGCTATGAT |
| PSC29475-1 | 3149 | Human | HMGB3 | NM_005342 | GGTGCAAAGGGTCCTGCTAAA |
| PSC29476-1 | 3149 | Human | HMGB3 | NM_005342 | GCCGTAATTGACACATCTCTT |
| PSC27079-1 | 10112 | Human | KIF20A | NM_005733 | GCCAGAAGAATATAAGGCTGT |
| PSC27080-1 | 10112 | Human | KIF20A | NM_005733 | GCAGTGCAAAGCAGAGCTAAA |
| PSC27081-1 | 10112 | Human | KIF20A | NM_005733 | CCGTTCCTGCATGATTGTCAA |
| PSC57463-1 | 9493 | Human | KIF23 | NM_004856 | GCCTTATTAGAACGTCAGAAA |
| PSC57464-1 | 9493 | Human | KIF23 | NM_004856 | GCAGAATAAACTCTGGGTTAA |
| PSC57465-1 | 9493 | Human | KIF23 | NM_004856 | CCCTTGGTCAATGACCTCATT |
| PSC45599-1 | 116372 | Human | LYPD1 | NM_144586 | AGCAAAGTGCCGGGATCATGT |
| PSC45600-1 | 116372 | Human | LYPD1 | NM_144586 | AGCATGAGAACACAGTAAATG |
| PSC45601-1 | 116372 | Human | LYPD1 | NM_144586 | CAGAAAGAAGTGATGGAGCAA |
| PSC45602-1 | 55388 | Human | MCM10 | NM_182751 | TCATCCTCAGAAGGTCTTAAT |
| PSC45603-1 | 55388 | Human | MCM10 | NM_182751 | GGGATAACTAGAGGTCAAATT |
| PSC45604-1 | 55388 | Human | MCM10 | NM_182751 | GACGGCGACGGTGAATCTTAT |
| PSC42495-1 | 23310 | Human | NCAPD3 | NM_015261 | CCCTCTGTGATTAGAGCACAT |
| PSC42496-11 | 23310 | Human | NCAPD3 | NM_015261 | CCTGATTAACAGTCCTACGTT |
| PSC42497-1 | 23310 | Human | NCAPD3 | NM_015261 | CGCTGGATCTTAGACTCGAAT |
| PSC29896-1 | 64151 | Human | NCAPG | NM_022346 | GCCTTAACAGTACATGACAAT |
| PSC29897-1 | 64151 | Human | NCAPG | NM_022346 | GAAGTCCACATAGAGAAGAAT |
| PSC29898-1 | 64151 | Human | NCAPG | NM_022346 | CGGGCAGTGTTATCATGTATT |
| PSC23271-1 | 55872 | Human | PBK | NM_018492 | CTCTTCTCTGTATGCACTAAT |
| PSC23272-3 | 55872 | Human | PBK | NM_018492 | GTCTGTGTCTTGCTATGGAAT |
| PSC23273-1 | 55872 | Human | PBK | NM_018492 | CACCAAGCAAATTATCAGAAA |
| psc14005-1 | 26147 | Human | PHF19 | NM_015651 | ccTGGCTAGCATATTTGACTT |
| psc14007-1 | 26147 | Human | PHF19 | NM_015651 | gcCACACATTTGAGAGCATCA |
| psc14008-1 | 26147 | Human | PHF19 | NM_015651 | ccCACCTCAAGTCATCTATCA |
| PSC45611-1 | 55771 | Human | PRR11 | NM_018304 | ATGAGTGGTAAACTTACAAAT |
| PSC45612-1 | 55771 | Human | PRR11 | NM_018304 | CACCAGAAAGAGTCGGTATTT |
| PSC45613-1 | 55771 | Human | PRR11 | NM_018304 | AGCCAAAGCCGAAAGATTATT |
| PSC46055-1 | 29893 | Human | PSMC3IP | NM_013290 | GAACATTAAAGCAGCTACCAA |
| PSC46056-1 | 29893 | Human | PSMC3IP | NM_013290 | CGGCAAGCAGAAGATCTATTT |
| PSC46057-1 | 29893 | Human | PSMC3IP | NM_013290 | GCAGAAAGAAATCCAGGAGTT |
| PSC27088-1 | 10615 | Human | SPAG5 | NM_006461 | CGCTCTGACAAGGAGTTAGAA |
| PSC27089-1 | 10615 | Human | SPAG5 | NM_006461 | CCATGCAACTGGATTATACAA |
| PSC27090-2 | 10615 | Human | SPAG5 | NM_006461 | CCAAATTAGCTCTACTCCTAA |
| PSC51237-1 | 147841 | Human | SPC24 | NM_182513 | CGTGTACGTGGCTCAACTTTA |
| PSC51238-1 | 147841 | Human | SPC24 | NM_182513 | TGCGAAGGAGCAGGTGCACCA |
| PSC51239-1 | 147841 | Human | SPC24 | NM_182513 | CGAGGACACGACAGTCACAAT |
| psc14084-1 | 10460 | Human | TACC3 | NM_006342 | gcTTGTGGAGTTCGATTTCTT |
| psc14085-1 | 10460 | Human | TACC3 | NM_006342 | ccAGGAAGTTCTGAGAACCAA |
| psc14086-1 | 10460 | Human | TACC3 | NM_006342 | gcAGTCCTTATACCTCAAGTT |
| PSC52163-1 | 55504 | Human | TNFRSF19 | NM_018647 | GCATCAACTCAGGATGCACTA |
| PSC52164-1 | 55504 | Human | TNFRSF19 | NM_018647 | GCTCAACGTCTTTGGATTCAA |
| PSC52165-1 | 55504 | Human | TNFRSF19 | NM_018647 | CATCTATTGTAAGAGACAGTT |
| PSC58109-1 | 3835 | Human | KIF22 | NM_007317 | CCTAGAGATTGAGAGGCTTAA |
| PSC58110-1 | 3835 | Human | KIF22 | NM_007317 | CCACCAGGAGACTCTCAAATA |
| PSC58111-1 | 3835 | Human | KIF22 | NM_007317 | CCTACAGAAGCTAAGCAGCAT |

**Appendix 6.** **The False Discovery Rate values for the selected set of genes.**

| **Gene** | **False Discovery Rate values** |
| --- | --- |
| PHF19 | 0.003426213 |
| DPYSL3 | 0.00917115 |
| CDC7 | 0.024618477 |
| FAM83D | 0.007150465 |
| FANCI | 0.007150465 |
| SPAG5 | 0.008925732 |
| FAM72A /// FAM72B /// FAM72D* | 0.016043657 |
| NCAPG | 0.020430822 |
| PSMC3IP | 0.031725354 |
| KIF23 | 0.039093878 |
| NCAPD3 | 0.019505251 |
| PRR11 | 0.006451895 |
| MCM10 | 0.022186093 |
| ZNF367 | 0.021110367 |
| KIF22 | 0.012303355 |
| LYPD1 | 0.006570987 |
| TNFRSF19 | 0.011490078 |
| PBK | 0.003426213 |
| FAM64A | 0.009410185 |
| HMGB3 | 0.006451895 |
| HAUS8 | 0.030344823 |
| CDCA5 | 0.00571916 |
| TACC3 | 0.005294533 |
| KIF20A | 0.003426213 |
| SPC24 | 0.012472511 |
| CIT /// MIR1178* | 0.012472511 |
| DSCC1 | 0.025743173 |

*FAM72A, FAM72B and FAM72D have high homology; and MIR1178 has high homology with CIT. Therefore, the same probe was used in these two groups respectively, and the detection data are consistent.

**Appendix 7.** **Details on shRNA sequences of** **selected genes.**

| **Gene** | **shRNA Virus number** |
| --- | --- |
| Ctrl | Negative control（Non-targeting shRNA） |
| KIF20A | PSC27079mix |
| HMGB3 | PSC29476mix |
| DSCC1 | PSC29887mix |
| LYPD1 | PSC45599mix |
| KIF23 | PSC57463mix |
| FAM83D | PSC57708mix |
| CDC7 | PSC14188mix |
| PBK | PSC23271mix |
| SPAG5 | PSC27088mix |
| NCAPG | PSC29896mix |
| NCAPD3 | PSC42495mix |
| MCM10 | PSC45602mix |
| PRR11 | PSC45611mix |
| PSMC3IP | PSC46055mix |
| SPC24 | PSC51237mix |
| TNFRSF19 | PSC52163mix |
| PHF19 | PSC14005mix |
| TACC3 | PSC14084mix |
| KIF22 | PSC58109mix |
