# Supplementary figures and images for: FAM83D acts as an oncogene by regulating cell cycle progression via multiple pathways in synovial sarcoma: a potential novel downstream target oncogene of anlotinib
Source: Discov Oncol. 2024 Mar 21;15:82. doi: 10.1007/s12672-024-00943-z (PMC10957831; doi:10.1007/s12672-024-00943-z)

Fig 3C FAM83D-1 WB


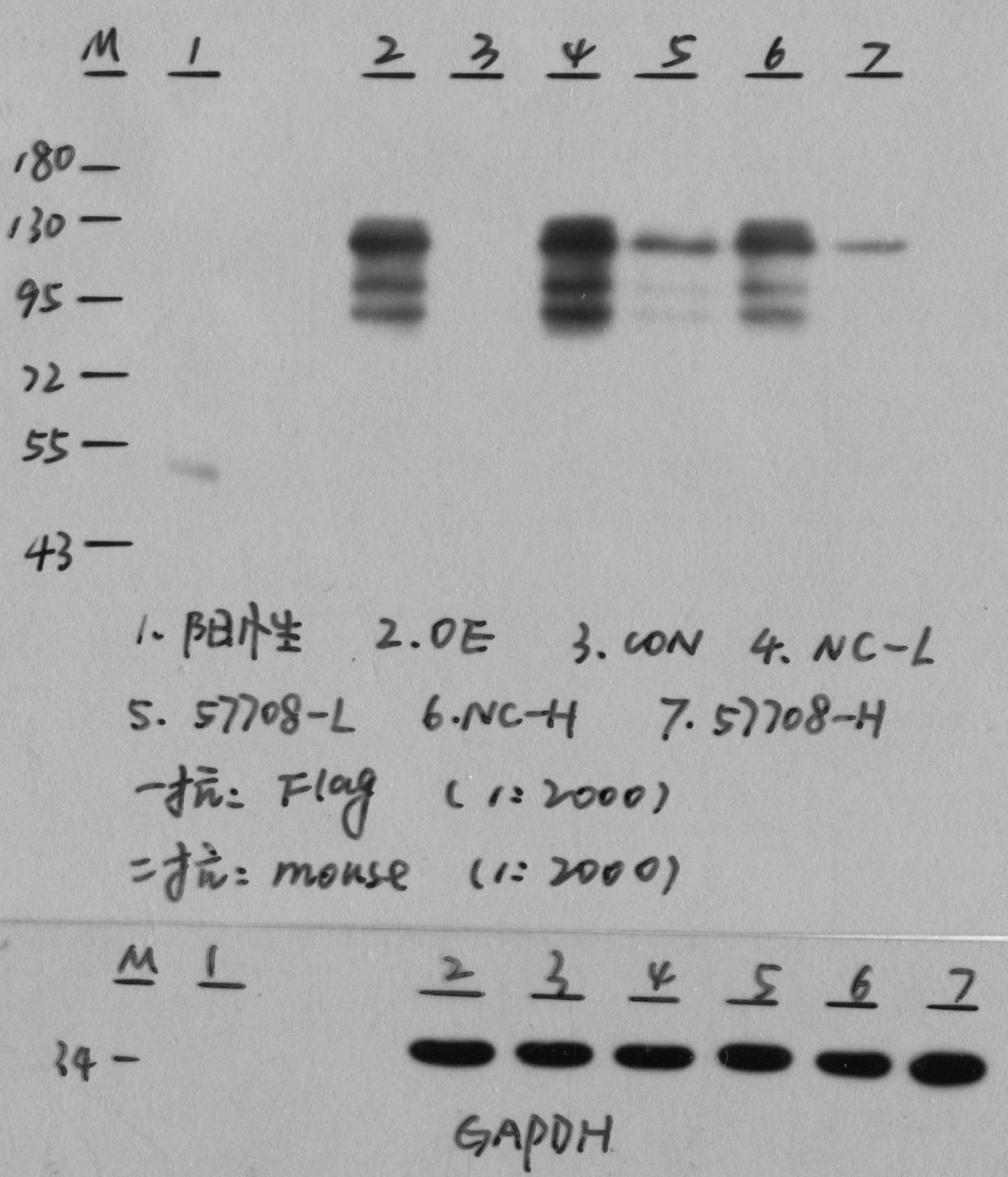


Fig 3C FAM83D-2 WB


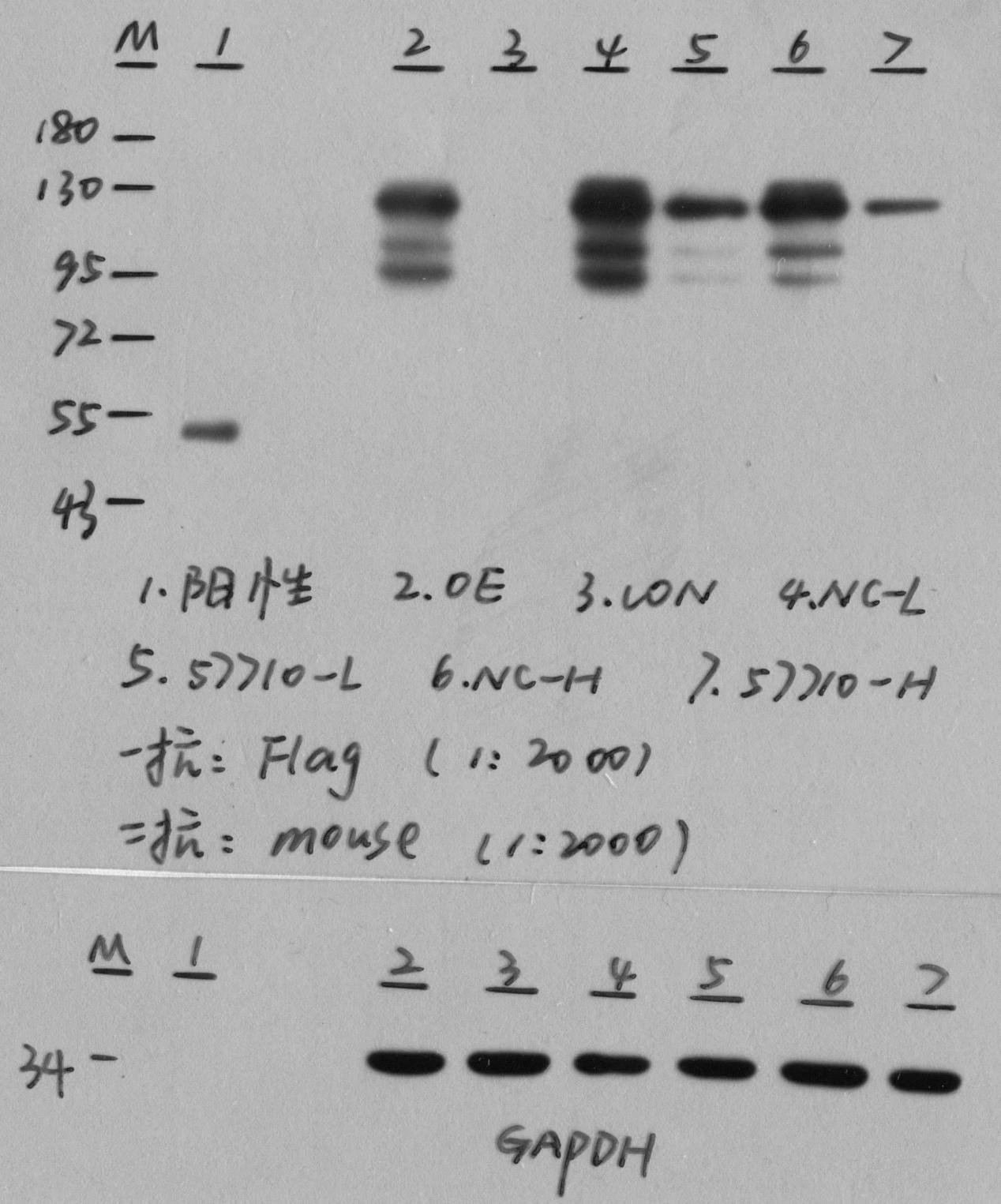


Fig 5F CDK1；BIRC5；GAPDH
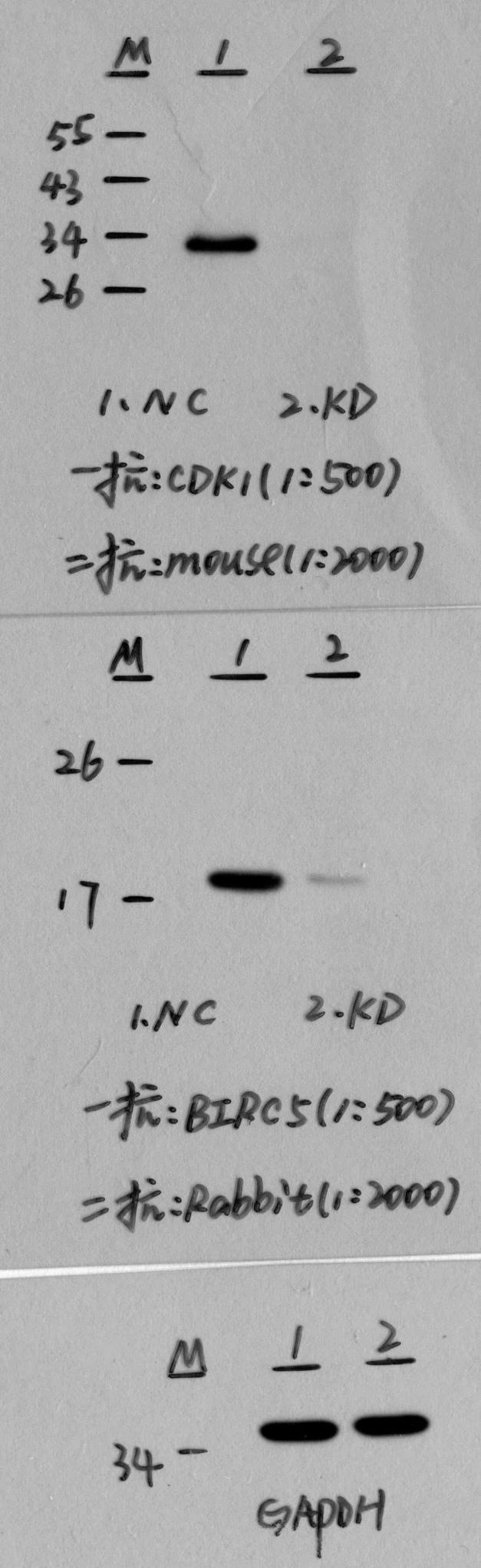


Fig 5F MCM2；CCNE1；GAPDH
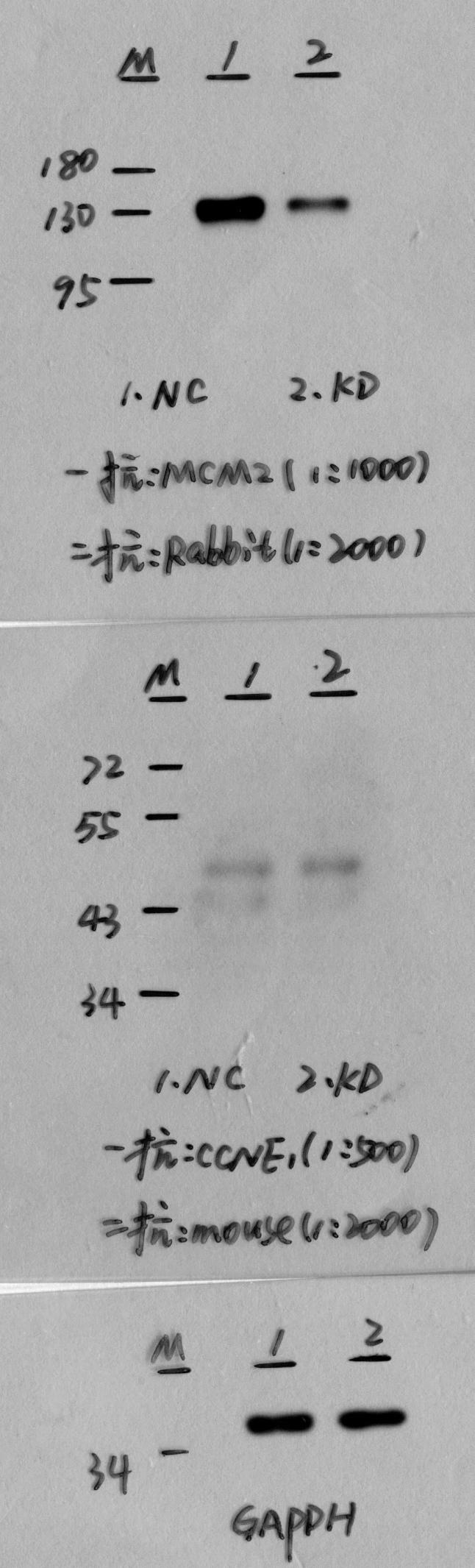


Fig 5F STAT1；GAPDH
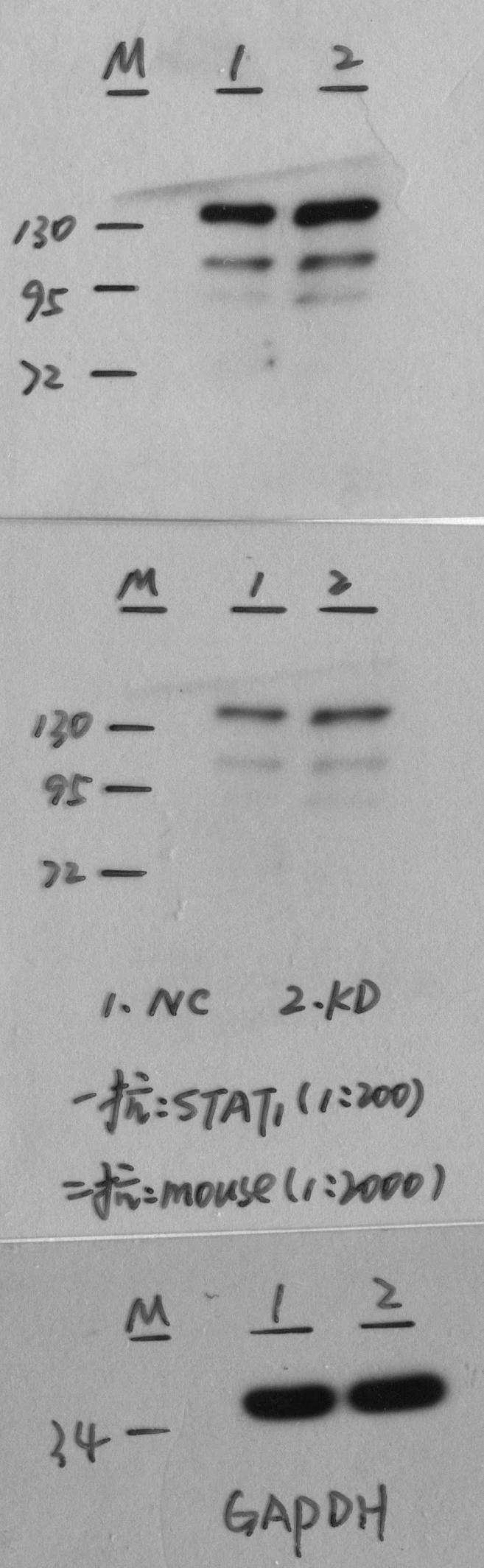

Supplement: Supplementary file 2 — (DOCX 796 KB) [file 12672_2024_943_MOESM2_ESM.docx]
